# Self-reported and routinely collected electronic healthcare resource-use data for trial-based economic evaluations: the current state of play in England and considerations for the future

**DOI:** 10.1186/s12874-018-0649-9

**Published:** 2019-01-09

**Authors:** Matthew Franklin, Joanna Thorn

**Affiliations:** 1 School of Health and Related Research (ScHARR), University of Sheffield West Court, 1 Mappin Street, Sheffield, S1 4DT UK; 2 School of Social and Community Medicine, University of Bristol Canynge Hall, 39 Whatley Road, Bristol, BS8 2PS UK

**Keywords:** Trial-based evaluation, Data collection methodology, Self-report, Routinely collected data, Large observational datasets, Big data

## Abstract

**Background:**

Randomised controlled trials (RCTs) are generally regarded as the “gold standard” for providing quantifiable evidence around the effectiveness and cost-effectiveness of new healthcare technologies. In order to perform the economic evaluations associated with RCTs, there is a need for accessible and good quality resource-use data; for the purpose of discussion here, data that best reflect the care received.

Traditionally, researchers have developed questionnaires for resource-use data collection. However, the evolution of routinely collected electronic data within care services provides new opportunities for collecting data without burdening patients or caregivers (e.g. clinicians). This paper describes the potential strengths and limitations of each data collection method and then discusses aspects for consideration before choosing which method to use.

**Main text:**

We describe electronic data sources (large observational datasets, commissioning data, and raw data extraction) that may be suitable data sources for informing clinical trials and the current status of self-reported instruments for measuring resource-use. We assess the methodological risks and benefits, and compare the two methodologies. We focus on healthcare resource-use; however, many of the considerations have relevance to clinical questions.

Patient self-report forms a pragmatic and cheap method that is largely under the control of the researcher. However, there are known issues with the validity of the data collected, loss to follow-up may be high, and questionnaires suffer from missing data. Routinely collected electronic data may be more accurate and more practical if large numbers of patients are involved. However, datasets often incur a cost and researchers are bound by the time for data approval and extraction by the data holders.

**Conclusions:**

Owing to the issues associated with electronic datasets, self-reported methods may currently be the preferred option. However, electronic hospital data are relatively more accessible, informative, standardised, and reliable. Therefore in trials where secondary care constitutes a major driver of patient care, detailed electronic data may be considered superior to self-reported methods; with the caveat of requiring data sharing agreements with third party providers and potentially time-consuming extraction periods. Self-reported methods will still be required when a ‘societal’ perspective (e.g. quantifying informal care) is desirable for the intended analysis.

**Electronic supplementary material:**

The online version of this article (10.1186/s12874-018-0649-9) contains supplementary material, which is available to authorized users.

## Background

Randomised controlled trials (RCTs) are generally regarded as the “gold standard” form of clinical trial for the purpose of providing quantifiable evidence around the effectiveness and cost-effectiveness of new healthcare technologies [1]. The importance of economic evaluations alongside clinical trials to assess cost-effectiveness has grown in recent decades, partly due to the need to show ‘value for money’ when investing in new care technologies and specific evidence requirements by reimbursement governing agencies such as the National Institute for Health and Care Excellence (NICE) in the United Kingdom (UK) [2], as an example. Within-trial economic evaluations rely largely on the acquisition of data describing the resource-use of trial participants (e.g. number of inpatient stays, outpatient visits, GP visits) to which unit costs are applied to provide cost data to inform the evaluation [3]. CH Ridyard and DA Hughes [4] conducted a systematic review of studies funded by the UK Health Technology Assessment (HTA) program to identify the different methods used for the collection of resource-use data within clinical trials; the majority of studies identified (61 of 85) used at least two methods, typically involving patient- or carer-completed forms and medical records (e.g. patient notes, large database), the latter of which is referred to as ‘routinely collected care data’ within this manuscript.

The aim of this paper is to compare the two aforementioned methods for collecting resource-use information for trial-based economic evaluations: (1) self-reported methods, whereby ‘self-report’ is by the patient about their own resource-use; (2) methods for using routinely collected resource-use data from electronic sources such as electronic versions of medical notes on administrative systems and large healthcare databases, which can contain information about individuals and cohorts of patients. We focus mainly on the acquisition of healthcare resource-use data (e.g. use of inpatient hospital care and seeing a nurse in a GP practice or home setting) rather than clinical outcome data (e.g. hospital anxiety and depression scale scores) as the focus here is specifically on data normally used for the purpose of economic evaluation; however, many of the considerations described also have relevance to other forms of clinical and health-related data which may be routinely collected and/or patient-reported. We also mainly focus on person-level data rather than aggregated data, because these are most desirable for trial-based evaluations. It should be noted that there are also objectively measured outcomes and investigator-reported data, which although may be routinely collected and used for clinical trials, are outside of the scope of this paper as these methods are rarely used to collect resource-use data.

The databases described within this manuscript are those which we, the authors, perceive to be the most popular used for the purpose of economic analyses; therefore, the databases described should not be considered a comprehensive list of all routinely collected data sources. We describe the potential strengths and limitations of each data collection method using specific databases as examples, and then discuss aspects for consideration before choosing which method to use when collecting resource-use data for trial-based evaluations. A compiled list of select websites is provided in Additional file 1: S1 to give the reader more information about the databases, software systems, and national Information Technology (IT) programmes mentioned in this paper.

### A note on information governance, data protection, and de-identified data

There are important data protection aspects to consider when using person-level data which are outside the scope of this paper; however a brief overview is provided in this section. It is important to note that, by law, National Health Service (NHS) Digital (the NHS’s internal IT provider), previously known as the Health and Social Care Information Centre (HSCIC), is the only organisation outside of direct care providers in the UK that can handle personal identifiable data (PID); however, licensed/approved organisations can also handle PID and trials are required to have explicit consent for PID. This paper focuses on person-level data for consenting people, so it is worth noting that self-reported and electronic data are governed by stringent information governance (IG) and data protection policies and regulations such as the General Data Protection Regulation (GDPR), which often requires de-identification (anonymisation, pseudo-anonymisation, and removal of person-identifiable data as required) of person-level data before it can be used for research purposes. Identification of people in electronic datasets normally requires explicit consent from patients included in studies for their NHS number to be used. Unique identifiers such as NHS number along with explicit consent are integral steps for using electronic data in most cases for trials (note, this might not be the case for observational studies using anonymised routine data); although, under GDPR, the legal basis for accessing such data is normally for research purposes, with consent alone no longer being the legal basis for accessing such data. These considerations are too complex to describe in detail here; however, these aspects will be discussed as something to consider when using either data collection method. Researchers should make enquiries about the necessary IG policies and data sharing agreements (DSA) as required.

### Main text

#### Overview: Use of self-reported and routinely collected care data

Traditionally, trial-based economic evaluations were largely reliant on self-reported methods for collecting resource-use information about the care resources people consumed. However, since the National Programme for IT, which ran from 2002 until 2013, prompted UK health and social care services to record person-level care-related data using Electronic Health Record (EHR) systems, there has been a keen interest in how to utilise these routinely collected data. These systems are usually designed by independent contractors, specifically for the administrative needs of the service while conforming to national specifications (e.g. general practitioner systems of choice [GP SoC] specifications [5]), and could provide a rich source of data (both resource use and clinical outcomes) for analysis in clinical trials rather than requesting similar data from the patient directly (e.g. how often the patient saw their GP or number of days spent as an inpatient). NHS Digital and other providers currently offer a variety of electronic databases for research and commissioning purposes. However, consenting trial participants may not have their data stored in these databases, data from any large database usually has both monetary and logistical costs, and the database may not contain appropriate Personal Identifiable Data (PID e.g. NHS numbers) to link data with participants. Owing to the complexities and cost of accessing data from routine sources, self-reported questionnaires are still commonly used to collect data for the purposes of research studies. Different types of resources may be collected using these two different methodologies; for example, hospital stays might be collected using routine data while informal carer arrangements, such as how much time a son cares for his elderly mother, would typically be collected by questionnaire because these data are probably not routinely collected. Some studies have used patient-report [4, 6, 7] and some have used a combination of patient-report data and collecting data from databases to provide complementary (i.e. each data collection method provides different information for the trial) or even substitutive (i.e. each data method provides similar information for cross-checking or validity assessment of the data obtained) information [8–10]. Even when administrative datasets are available, there are conflicting reports of the validity of routinely collected data compared with patient-reported data even when the data collected is perceived to be based on the same type of resource-use [11]. Therefore, neither data collection method can currently be considered the ‘gold standard’ for the purpose of informing trial-based analyses; rather, it is up the researcher to decide on which method to use dependent on the circumstance (e.g. trial design, setting, aims and objectives) and the data of interest for the trial.

#### Self-reported methods

Many trials employ data-collection methods based on patient recall [4], in part because a questionnaire is relatively cheap. Use of questionnaires allows the researcher to control data collection to a large degree, as it does not rely on third parties granting access; the value of this element of control should not be underestimated in trial situations with significant time pressures. Researchers can also tailor questionnaires to request specific information needed for analysis. This is particularly important when an economic analysis from the societal perspective is planned, as data on patients’ productivity, travel methods, and informal care or childcare requirements, for example, can only come from the patient.

However, relying on patient recall has some significant disadvantages. Patients tend not to be able to recall their resource use accurately [12] and this gets worse as the time period over which they must remember increases (e.g. retrospectively remembering the care they have received over 3 or 6 month periods); if the inaccuracy is systematically different between trial arms, it can constitute a recall bias, leading to biased estimates. Data entry clerks can introduce mistakes when capturing data electronically from paper-based questionnaires, either by simple typographical error or by reinterpreting non-standard responses in an arbitrary fashion. Completing questionnaires is time consuming for patients; researchers (and ethics committees) prefer to minimise the research burden placed on patients and primary investigators are often keen to minimise the amount of space taken up by resource-use questions. Although adjusting for baseline differences in resource-use is recommended as part of statistical analyses associated with trials [13], collecting data by patient recall at baseline involves an additional burden on patients, and is typically not undertaken. As a result of many of these issues, resource-use questionnaires tend to suffer from missing data [14].

In terms of the practical development and administration of questionnaires, there is a significant amount of research time wastage. These instruments are rarely validated [4], and even more rarely re-validated when used with alterations or in a different context. Reporting also tends to be poor [7]. As a partial solution to some of these problems, the Database of Instruments for Resource-Use Measurement (DIRUM) was created as a repository for instruments based on patient recall (www.dirum.org). It is a useful source for researchers wanting to find an instrument that may be reusable in their own work and the database is recommended for use by the International Society for Pharmacoeconomics and Outcomes Research (ISPOR) taskforce on good research practices [15].

Standardisation of patient-reported outcome measurement in economic evaluation has been accepted as a principle for some time, with NICE recommending the use of the EuroQol five-dimension (EQ-5D) instrument [16, 17] as a measure of preference-weighted health status [2] to enable cross-comparison of outcomes across trials. Standardised instruments for patient-reported cost measurement have also been developed, with the Client Service Receipt Inventory (CSRI) the most commonly used and validated example [18]. However, the CSRI was originally developed to be administered as an interview, which is a costly means of gathering data and impractical in many trials. It has also been adapted many times, with over 200 versions believed to have been used [19], and was created specifically for use with patients with mental health conditions. It is therefore neither fully standardised nor universally applicable. The Annotated Patient Cost Questionnaire (APCQ) [20] was a more recent attempt to standardise cost measurement, and has invoked much interest since being deposited on DIRUM. However, it requires some effort to use effectively and, although it appears to perform well in validation studies [21], has not yet been widely adopted. Other standardised resource-use questionnaires have been developed, including one in Dutch [22], one for cancer trials [23], and one for patients with dementia [24], but a standardised (and well validated) instrument that is relevant to all trials and could be used ‘off the shelf’ by researchers in the UK is lacking. While this ideal may not be fully achievable, a recent Delphi survey based on the opinions of health economists with experience of UK-based trials found that it was possible to identify a list of 10 essential items that should be included in such a standardised instrument [25], with additional modules identified as important in some cases.

#### Electronic data sources of routinely collected data

Within this section we explore the use of routinely collected data; from raw data extracted straight from the service up to the level of linked datasets across multiple services, and the evolution of more efficient trial designs utilising these data.

#### Raw data extraction

Raw data extraction can include anything from direct system data extraction and anonymization to recording data from computer screens into data extraction forms. Of 63 studies that used patient-reported methods, 43 supplemented these data with routine sources such as GP records, hospital notes, and hospital databases [4]. A previous study by M Franklin, V Berdunov, et al. [26] extracted raw data from a range of services, including hospitals’ Patient Administration Systems (PAS), primary care practice EHR software systems (including SystmOne [27], EMIS [28], and Vision [29]), as well as from the electronic systems of intermediate care, mental health trust, ambulance, and social care services; these methods were then used to obtain data for two subsequent RCTs [30, 31]. It is worth noting that when obtaining primary care data and other external service data (e.g. community care), SystmOne proved particularly useful within these studies because remote access was possible due to the central database and interoperability of this system – information pertaining to patients’ care from any SystmOne system can be held centrally and then accessed providing a smart card with appropriate permissions from the practice has been gained (and the person and service have consented to certain data access) [26, 32, 33]. However, the permissions and time required to obtain data from all these disaggregated services and their electronic systems makes obtaining such data labour intensive and so not viable for many studies.

Primary care data have been particularly disaggregated among a number of software systems (e.g. EMIS, SystmOne, Vision) [34] and clinical coding systems (e.g. Clinical Terms Version [CTV3] and Read Version 2) which has caused issues for researchers. MIQUEST was an example of a stand-alone query language within GP practices that could be used to extract certain data parameters; however, the output from such a query language limited its use for economic analysis despite its benefits for patient identification based on characterising patients using Read codes (such as if patients with a particular condition need to be identified for a study, for example) [35–38]. A current move to the Systematized Nomenclature of Medicine - Clinical Terms (SNOMED CT) as a single clinical terminology within primary care [39] and across other care settings [40] could deal with some cross-system data extraction issues, but this will need to be examined in future research; it should also be noted that due to the implementation of SNOMED CT, NHS Digital announced that they would not be undertaking any future development work on MIQUEST (as it was not compatible with SNOMED CT) and advised that organisations plan to transition away from MIQUEST by April 2018 [41, 42]. Attempts to enable better formats for extracted information from primary care software systems, such as the Apollo software system [10, 43, 44], could enable trial-based analysis and should be assessed in future research.

Raw data collection often requires aid from data processors or those with knowledge of health informatics at the service, or researchers with appropriate knowledge, training and permission to access and process the data. This imposes a practical restriction on researchers using the systems as they must liaise with third parties.

#### NHS digital and commissioning datasets

NHS Digital provides a variety of electronic datasets (see Table 1 for examples), offering access through their Data Access and Request Service (DARS) dependent on a five-stage process (application, approval, access, audit, and deletion). NHS Digital have appropriate permissions to handle PID and therefore accessing data for consenting patients is in theory feasible assuming information governance (IG) and data-sharing agreements can be arranged; this may not be possible with other datasets (see section titled "Other large observational datasets: Primary care").

**Table 1:** An overview and summary of some potential electronic databases for resource-use information; please note that the list is not exhaustive

Name of database/ service software	Service category	Comments about the data	Data dictionary (Yes/No)
Primary care databases			
Clinical Practice Research Datalink (CPRD)	Primary care	Collects data from Vision (historically), EMIS (more recently), and potentially SystmOne (being piloted at time of writing) GP practice software systems. Reportedly covers over 11.3 million patients (4.4 million active patients) from 674 practices in the UK ^b^ – this was the figure reported for just Vision practices.	Yes (Read code based)
The Health Improvement Network (THIN) database	Primary care	Collects data from Vision GP practice software systems. Reportedly covers 11.1 million patients (3.7 million active patients) from 562 general practices in the UK ^b^	Yes (Read code based)
ResearchOne	Primary care (and other contributing organisations – see ‘comments’)	Collects data from SystmOne GP practice software systems. Also reportedly collects data from other services using SystmOne. As of 2013, ResearchOne reportedly includes 5 million health records from 400 contributing organisations across 10 organisation types (ranging from hospitals to end-of-life organisations)^b^	Yes (Read code based)
QResearch	Primary care	Collects data from EMIS GP practice software systems. Based on current publications associated with this dataset, unsure what resource-use data is available as not used for economic studies. As of 2015, the database reportedly obtains data from a sample of approximately 1000 practices covering a population of 18 million people^b^	Yes (Read code based)
NHS Digital databases			
Secondary Uses Service (SUS)	Healthcare data	Designed to provide anonymous patient-based data for purposes other than direct clinical care, such as healthcare planning, commissioning, public health, clinical audit and governance, benchmarking, performance improvement, medical research and national policy development. SUS will only provide data for the region of interest to the commissioners if obtained through NHS commissioners. SUS is updated once a month.	Yes (note, includes a variety of commissioning data)^d^
Hospital Episode Statistics (HES)	Secondary care	Hospital care data (inpatient, outpatient, A&E, and critical care). Once a month and at pre-arranged dates during the year, SUS takes an extract from their database and sends it to HES – it is this data which populates the HES database.	Yes (online)^e^
General Practice Extraction Service (GPES)	Primary care	GPES is part of the GP collection service alongside the Calculating Quality Reporting Service (CQRS) used to record practice participation and to process and display information. GPES collects primary care information from GP IT systems and then presents it at a National level. Used to inform GP payments. Collects both anonymised and person-identifiable data (PID; when permitted). Main focus is clinical data (i.e. Quality Outcomes Framework [QOF] data), not resource-use data.	General overview of data that can be viewed in GPES is listed online^f^
Diagnostic Imaging Dataset (DIDS)	Diagnostic	NHS-funded diagnostic imaging tests	Yes (online)^g^
Improving Access to Psychological Therapies (IAPT)	Mental health	Adults in receipt of NHS-funded IAPT services (see data dictionary).	Yes (online)^h^
Mental Health Services Data Set (MHSDS^b^	Mental health	Record-level data on care of children, young people and adults who are in contact with mental health, learning disability or autism spectrum disorder services.	Yes (online)^i^
Raw data extraction based on study by M Franklin, V Berdunov, et al (2014)^a^			
Patient Administration System (PAS)	Hospital	Hospitals collect data into PAS (in Sheffield this is the Lorenzo system, which is a well-established system in England). This dataset includes basic hospital activity (i.e. inpatient, outpatient, and A&E); other detailed clinical information may be held on other hospital systems.	See HES data dictionary for a general overview
SystmOne, EMIS, Vision	Primary care	SystmOne, EMIS and Vision currently dominant primary care software systems in England. Each collects and records data slightly differently, but underlying data are coded based on Read Codes (most if not all should be using SNOMED CT by April 2018). Note, the methods used by Franklin et al (2014) did not rely on the use of Read Codes, rather front-end report outputs which were processed using visual basic for application (VBA) scripts.	See ‘Read Codes and SNOMED CT’
Intermediate care, mental health trust, ambulance services, and social care systems	Various services	The study by M Franklin, V Berdunov et al (2014) collected raw electronic data from all these systems. It is possible to collect these data after discussion with the service and consent agreements from the patients of interest.	No – data based on discussion with services
Technology for future consideration			
Read Codes and SNOMED CT	Primary care	Read codes can be obtained from the Technology Reference data Update Distribution (TRUD) website. SNOMED CT is a more unified coding base than current Read codes. Software systems have been developed to export information from primary care systems in a more usable manner, such as the Apollo software system.	Yes – a Read Browser and Read codes are required ^j^
GP Connect and Data Commissioning Flows	Primary care (initially)	GP Connect and Data Commissioning Flows works are in their early stages; it is difficult to gauge the possible benefits these plans will bring from a researcher perspective.	N/A
Bespoke linked dataset examples			
NorthWest EHealth linked database	Linked datasets	Information on medications, symptoms and use of healthcare facilities.	Contact provider
CALIBER dataset	Linked datasets	Linked data for primary care (CPRD), coded hospital records (HES), social deprivation information and cause-specific mortality data (ONS).	Contact provider

Footnote. Website references for all of the aforementioned databases and software systems are provided in Box 2. Links to online data dictionaries, if available, are listed at the end of this footnote

^a^These figures were reported on the databases own website and were still present on the website as of 10th April 2017

^b^Resource-use data could be a part of any database in theory because some of these aspects are coded in the services; however, they are only useful if the data are then included in the larger databases at the person-level (rather than national level, such as for GPES)

^c^Note, based on comments from the NHS Digital Standardisation Committee for Care Information (SCCI) in 2015(https://nhs-digital.citizenspace.com/scci/mhsds/), MHSDS is a change to the Mental Health and Learning Disabilities Data Set (MHLDDS) that supersedes and replaces this standard, as well as the following: Child and Adolescent Mental Health Services (CAMHS) data set; Mental Health Care Cluster; Mental Health Clustering Tool; Learning Disabilities Census (LDC), included in Assuring Transformation standard. MHSDS will also incorporate the requirements of Children and Young People’s Improving Access to Psychological Therapies (CYP IAPT)

^d^SUS data dictionary (includes a variety of Commissioning Data Sets [CDS]): http://www.datadictionary.nhs.uk/data_dictionary/nhs_business_definitions/s/secondary_uses_service_wu.asp?shownav=1

^e^ HES data dictionary: https://digital.nhs.uk/data-and-information/data-tools-and-services/data-services/hospital-episode-statistics/hospital-episode-statistics-data-dictionary

^f^ CQRS services and data that can be viewed in GPES: https://digital.nhs.uk/services/general-practice-extraction-service#types-of-data

^g^ DIDS data dictionary: http://www.datadictionary.nhs.uk/data_dictionary/messages/clinical_data_sets/data_sets/diagnostic_imaging_data_set_fr.asp?shownav=1

^h^ IAPT data dictionary: http://www.datadictionary.nhs.uk/data_dictionary/messages/clinical_data_sets/data_sets/improving_access_to_psychological_therapies_data_set_fr.asp?shownav=1

^i^ MHSDS data dictionary: http://www.datadictionary.nhs.uk/data_dictionary/messages/clinical_data_sets/data_sets/mental_health_services_data_set_fr.asp?shownav=1

^j^ Read codes (https://isd.digital.nhs.uk/trud3/user/guest/group/2/pack/9) and SNOMED CT UK edition (https://isd.digital.nhs.uk/trud3/user/guest/group/2/pack/26)

NHS Digital’s Hospital Episode Statistics (HES) dataset has become popular for the purpose of analysis within England. HES provides a large amount of aggregated or person-level hospital care data and unified codes, such as: International Classification of Diseases version 10 (ICD-10); Office of Population and Surveys Censuses (OPCS) Classification of Interventions and Procedures version 4 (OPCS-4) codes; and Healthcare Resource Group (HRG) codes which can be linked with reference cost data [45, 46]. However, note that HES only has HRG codes until 2012; researchers will need to use HRG software toolkits to derive HRGs for the latest HRG version 4+ (HRG-4+) [47, 48] and earlier versions (e.g. HRG-4 and HRG v3.5) [48] from codes within HES.

In addition to HES, NHS Digital provides a variety of electronic datasets including:

- Secondary Uses Service (SUS) [49] (note, HES comes from SUS);
- General Practice Extraction Service (GPES) [50] (note, part of the GP collections service [51] alongside the Calculating Quality Reporting Service [CQRS] to record practice participation and to process and display information);
- Diagnostic Imaging Dataset (DIDS) [52];
- Improving Access to Psychological Services (IAPT) [53];
- Mental Health Minimum Data Set (MHMDS) [54].

Not all the data provided in these datasets are useful for the purpose of clinical or economic evaluations, but they are useful examples for indicating the amount of electronic data available. Descriptions of these databases and links to their data dictionaries are provided in Table 1. It is also worth noting that various mental health datasets have or may be amalgamated into the Mental Health Services Data Set (MHSDS) [55] (see also Table 1).

NHS Digital also provides datasets for commissioners, such as the current Clinical Commissioning Groups (CCGs: clinician-led bodies responsible for commissioning healthcare services within a local area). The data flows via the Data Services for Commissioners Regional Offices (DSCROs), who provide data to a CCG for their geographical area of interest and responsibility only. The CCG may also have ‘local data’ flows such as from acute, primary, mental health, and social care services to supplement NHS Digital data. Partnerships between commissioners and researchers can benefit both parties and patients through the research objectives and outcomes that could be achieved by sharing data. However, these datasets are governed by legislation and, often, NHS Digital policies; the CCG are bound by the terms of their data sharing agreement with NHS Digital.

#### Other large observational datasets: Primary care

For primary care data, a variety of datasets exist (which obtain their data from specific GP software systems) including: Clinical Practice Research Datalink (CPRD; traditionally obtains its data from the Vision system, although has reportedly started obtaining data from practices with the EMIS system and is piloting to obtain data from SystmOne) [56, 57]; The Health Improvement Network (THIN; Vision) [58]; ResearchOne (SystmOne) [59]; and QResearch (EMIS) [60].

However, these primary care databases do not allow access to PID (e.g. NHS number) and therefore are not suitable for existing trial-based evaluations with consenting patients. Note that although PID cannot be accessed through these databases, the database providers may be able to extract data from primary care systems for consenting patients (for example, ResearchOne explicitly offer such a service on their website); there is also still potential to develop more efficient study designs using databases such as these (e.g. CPRD) while maintaining the anonymised nature of the data (e.g. no PID; see section titled "Efficient study designs using large observational datasets"). It is worth noting that L McDonnell, B Delaney and F Sullivan [61] have compiled a more comprehensive list of UK primary care datasets that may be of interest.

#### Linked datasets

An issue with electronic datasets is that they may only adequately record data for patients who use that particular service. Previous studies have already described the need for linked data when assessing the burden of injury [62] and for myocardial infarction [63]. This aspect has also been raised as a potential issue when interpreting information about external care services (e.g. hospital and community services) recorded within primary care [64, 65]. There have been attempts at linking datasets, for example: (i) Hospital Episode Statistics (HES) has data linkages with the Office for National Statistics (ONS) mortality data (including causes of death), PROMs (patient reported outcome measures), diagnostic imaging, and MHMDS datasets; (ii) a subset (approximately 75%) of consenting CPRD English practice data can be linked with HES, ONS (mortality data), area-based deprivation data, and Cancer Registry UK data [64].

Other examples of linked datasets described in Table 1 include the (i) NorthWest EHealth linked database and (ii) Clinical research using LInked Bespoke studies and Electronic health Records (CALIBER) dataset. For the latter, the practical issues with using linked data have been described by M Asaria, K Grasic and S Walker [66]. Linking datasets is important to obtain the best possible data for the services of interest and is an aspect for consideration if the proposed analysis requires a more holistic approach rather than focussing on a single service.

#### Efficient study designs using large observational datasets

There is currently a move to enable RCTs to be carried out in large databases of routinely collected data while retaining the anonymised nature of the data – these have been described as ‘efficient’ [65] and ‘pragmatic’ [67] study designs. Using data from existing patient groups within these large observational datasets has been suggested as a way to enable patients to be entered into RCTs more quickly than traditional study designs [67]; however, without patient consent, these data must be kept anonymised for research purposes. CPRD have designed methods for interventional research to take place within the confines of their anonymised dataset by enabling practices to recruit patients and then randomise them to a treatment before data are then entered into the EHR records and anonymised as normal [68]; an example of a cluster RCT and economic evaluation within CPRD is the PLEASANT trial [65, 69, 70]. Another example of an efficient study design using similar methods includes the Salford Lung Study [44] using the NorthWest EHealth linked database. Although these trial designs are not currently the norm, they represent a methodology which may enable trials and the accompanying analyses to utilise routinely collected electronic data; however, such trials are constrained to rely only on the data routinely collected within the associated large database, meaning it may not be viable for all trials dependent on the relevant trial outcomes and associated data requirements.

## Discussion

In theory, EHRs should provide more detailed and accurate resource-use data than self-report, as these databases are meant to represent the care patients have actually received. There are also factors affecting the accuracy of self-reported data including simple recall errors, or a patient’s lack of knowledge about care structures (potentially leading to appointments with nurses being reported as doctors, for example). As such there seems to be a push towards using electronic databases of routinely collected data rather than self-reported methods, although relieving the burden on patients receiving care is also a major driver for using these databases. There is also a costing perspective argument that because electronic data inform reimbursement, such as through payment by results (PbR) [71], these are the data that should be used as part of an economic evaluation. However, not all electronic datasets are designed to be used as a data source for clinical trials and there is “cautious” support of the exchangeability of both self-reported and administrative resource-use measurement methods in the empirical literature [72–74]. In a European Delphi study to produce consensus-based, cross-European recommendations for the identification, measurement and valuation of costs in health economic evaluations, the recommendation was to use patient-report for measuring resource use (and lost productivity) over electronic sources, such as large databases, as such databases don’t cover all necessary care services [75].

For hospital data, there has been an attempt at unified data processing and coding. Inpatient and outpatient data from HES are reasonably valid for research [76], although clinician engagement has been questioned [77] and time delays for obtaining HES data have been a concern for researchers working to tight study time horizons. In GP records, negative systematic bias has been demonstrated with patients consistently reporting higher numbers of non-GP contacts than GP records; this questions the reliability of primary care systems for recording external care data [73]. Such reliability concerns have been used as rationalisations for needing linked datasets [62, 63, 65]. Current proposed innovations to enable better interoperability (and potentially better linked data) includes GP Connect [78]. The long term vision of GP Connect is to enable interoperability between systems, with an initial idea to develop this link using the Digital Interoperability Platform, an NHS Digital Spine service [79]. NHS England are also planning some National Commissioning Flows work to streamline the flow of healthcare data: one aspect of this work is to improve national datasets and to drive improvements to standardised data definitions [80]. These innovations could improve data flows and therefore improve access and connectivity between different services and their datasets to improve patient care and assessments, perhaps eventually moving to fully integrated care records. However, historically, national attempts at linking electronic care systems have not been able to achieve fully integrated care records, with key reasons being attributed to poor progress with intended deliverables associated with unnecessary costs/spending in an evolving IT system for the National Programme of IT [81–83], and a perceived lack of data security/patient data protection for the care.data programme [84]. As such, information governance (IG) evolves and accessing electronic person-level data for research becomes too complicated and time-consuming. A previous study has categorised the challenges of accessing routinely collected data from multiple providers in the UK for primary studies into five themes [85]: data application process; project timelines; dependencies and considerations related to consent; information governance; contractual. Such challenges are difficult, time consuming, and even costly to overcome; therefore, electronic data methods may be pushed to the side in favour of using and refining simpler, self-reported methods.

The literature on questionnaire development is extensive and improvements could be made by following evidence-based guidelines [86]. However, a number of questions surrounding best practice remain. For example, the optimum recall period has not been established [87]; recalling salient events such as hospital admissions over a year may be adequately accurate for the general population (perhaps less so for people with cognitive impairment or people receiving integrated care across multiple services), whilst commonplace events such as GP appointments may require a much shorter recall time period. The scope of the questions can lead to problems for patients identifying relevant events; for example, if a patient is asked to report only resource-use relevant to their diabetes, they may struggle to correctly identify that a fall is relevant.

Social care data are not discussed in this paper because of the nature of the systems used. Healthcare systems are more usable for obtaining data relative to social care systems because of aspects such as the inclusion of unique identifiers (NHS number or other pseudo codes), relatively more standardised coded data, established national data dictionaries, and national software and system requirement (e.g. GP SOC). A structured discussion around social care data would be more complex because of the lack of structure of social care systems; however, social care is an area which requires consideration in the future.

Based on this discussion, there are a number of key elements a researcher may want to consider before deciding on a data-collection method alongside an RCT or any clinical trial which requires patient-level data (see Table 2).

**Table 2:** Aspects to consider when choosing to obtain resource-use data using self-reported or electronic methods

Aspects to consider	Self-reported	Electronic database
Access to person-level or record-level data	Data reported by the patient themselves (or a proxy on their behalf) are patient-level by definition.	Currently a major issue for electronic datasets. To those without advanced knowledge of large datasets, it is unclear whether person-level data can be obtained and the IG aspects for obtaining these data are challenging for researchers. There may also be a restricted data flow of person-level data depending on the current stance of the data holders of what constitutes appropriate data protection policies (e.g. NHS Digital)
Service for which data are required	Essential for services with no electronic records; for example, travel, childcare, over-the-counter medications	All care services should operate an electronic administrative system from which data could be obtained – will only collect data based on care service provided or if linked to another service (e.g. CPRD linked to HES; SystmOne central database).
Practicality and cost	Pragmatic and cheap method which is well understood and largely under the control of the researcher	Large datasets often incur a cost and the researcher is bound by the time for data approval and extraction by the data holders. Raw data extraction can be time consuming and relatively costly compared with self-reported methods.
Number of patients	Administratively burdensome for large numbers of patients	If a large dataset exists and contains some person-level identifier code (e.g. NHS number), then obtaining data for large patient numbers is possible. For raw data extraction, less practical for large numbers of patients unless a systematic method for data extraction is available (e.g. software system for data extraction).
Validity of data	Known issues with validity of self-reported data, particularly problematic if differential between arms. Can be tested in a pilot phase.	Large databases have been known to validate their data; however, the extent to which these data are validated is not transparent, and validity for costing purposes may not have been tested. Raw data are complicated to validate.
Time horizon for analysis	Loss to follow-up may be higher with a lengthy time horizon. Self-reported methods may work better for shorter time horizons (i.e. one questionnaire per 3 month time period of interest).	Depends on time horizon of the database. Loss to follow-up can occur in large datasets and raw data depending on the database or service (e.g. GP practice may change system restricting eligibility to provide data to particular primary care datasets).
Patient group being analysed	Care may be needed with particular patient groups who lack capacity, for example	Different patient groups may use different services from which data may need to be obtained. Type of patient (e.g. cognitive ability) is not generally a concern.
Type of costing exercise (e.g. top-down or micro-costing)	Can be tailored exactly to the type of costing exercise required but depends on knowledge of patient to provide the detail of care consumed. More time consuming collecting detailed information for micro-costing exercises.	Raw and large datasets can offer aggregated or very detailed information based on the level of data recording. Some data offered may still not be reliable for micro-costing (e.g. time with patient recorded in large databases such as CPRD).
Recall bias	Problematic if differential recall errors exist systematically between arms of a trial	Recall bias is not an issue, but potential bias relies on accurate data recording at the service-level.
Missing data	A known problem with self-report; can be minimised by following good practice	Missing data is not a ‘known’ issue – if data are missing, then not easy to assess (i.e. it would be assumed there was no resource-use). Some evidence of data missing from HES, but would be difficult to assess extent in a trial.
Regional or national study	Data can be collected consistently across geographical areas	More detailed datasets are available regionally than nationally. National datasets depend on service uptake to provide electronic data. Raw data may be difficult to obtain electronically if there is no remote access to the software system (e.g. remote access is possible with SystmOne).
International studies (outside of England)	Self-reported data is still necessary for many countries and necessary in circumstances where electronic systems are not available or cannot provide the data required.	More countries are using electronic data provided by care services, commissioners, and insurance companies (to name a few sources). This is important to note when comparing analysis in England with other international studies. Comparably, this may limit our (i.e. studies based in England) ability to perform the best possible analysis which is desirable as part of research studies.
All-cause or disease specific assessment	Patients may struggle to correctly identify whether an event is related to their condition or not	A variety of codes (e.g. ICD-10 and OPCS-4 for in-hospital codes) and free text to specify whether resource-use is associated with a condition. Primary care data has Read or SNOMED CT codes for specific conditions and diseases, although these codes are not always used appropriately. Free text is difficult to use. HES outpatient diagnosis codes are poorly completed.
Baseline measurements	Additional burden on patient and very rarely collected.	Not an issue if the data are available for the baseline period of interest.
Experience and familiarity	Relatively easy for a researcher to get up to speed with. Design for a clinical study may require knowledge of the clinical area to accurately collect the resource-use cost drivers.	For large datasets, requires a data requisition form to be completed which is not always easily understood. For commissioning data, requires a contact with access to the data and a data requisition form to be completed. For raw data, requires knowledge of the service or to identify a person who can extract the data (i.e. trained researcher of practice nurse).
Information Governance	Managed through standard ethics application methods.	IG is a major concern when using electronic data. This process can be navigated with expert guidance, although the developing world of electronic data will always be a concern for researchers.
Social care data	Social care data could be self-reported and the exact type of social care data of interest could be specified within the questionnaire.	Routinely collected social care data is not discussed in this paper, but is an important aspect for future consideration. Healthcare systems are more usable for obtaining data relative to social care systems because of aspects such as the inclusion of unique identifiers (NHS number of other pseudo codes), relatively more standardised coded data, established national data dictionaries, and national software and system requirement.

## Conclusions

Until electronic databases become more integrated across care services and more reliable in terms of data processing and extraction alongside tight time restrictions, self-reported methods will be used for collecting resource-use information and electronic data will remain an underutilised resource alongside trials. Hospital data are relatively unified and offer parameters useful for clinical and economic analysis. Generally, hospital care constitutes a major driver for patient care; therefore, the detailed electronic data should be considered superior to self-reported methods if hospital data are the main focus of the analysis (with the caveat of requiring data sharing agreements with third party providers and potentially time-consuming extraction periods). However, for all other resource-use, self-reported methods may be the preferred option given the current complications around electronic data.

## Additional file

Additional file 1: **Appendix S1.** “Relevant websites for further information”: this supplementary appendix includes websites (URLs) to complement or supplement discussion points within the manuscript to aid the reader learn more about the databases, software systems, and national IT programmes. All websites were accessed as of 23rd August 2017. (DOCX 19 kb)
