# Supplementary material for: Self-reported and routinely collected electronic healthcare resource-use data for trial-based economic evaluations: the current state of play in England and considerations for the future
Source: BMC Med Res Methodol. 2019 Jan 9;19:8. doi: 10.1186/s12874-018-0649-9 (PMC6325715; doi:10.1186/s12874-018-0649-9)
Supplement: Supplementary file 1 — Appendix S1. “Relevant websites for further information”: this supplementary appendix includes websites (URLs) to complement or supplement discussion points within the manuscript to aid the reader learn more about the databases, software systems, and national IT programmes. All websites were accessed as of 23rd August 2017. (DOCX 19 kb) [file 12874_2018_649_MOESM1_ESM.docx]

**Supplementary Appendix S1.** **Relevant websites for further information**

The below presents, in alphabetical order, websites (URLs) to complement or supplement discussion points within the paper to aid the reader learn more about the databases, software systems, and national IT programmes. All websites were accessed as of 23^rd^ August 2017.

1. Apollo Data Extraction: turning data into information [<http://www.apollo-medical.com/apollo-sql-suite/>]
2. CALIBER: Clinical research using LInked Bespoke studies and Electronic health Records [<https://www.ucl.ac.uk/health-informatics/caliber>]
3. Review of health and care data security and consent: care.data [https://www.gov.uk/government/speeches/review-of-health-and-care-data-security-and-consent]
4. Clinical Practice Research Datalink (CPRD) [<https://www.cprd.com/intro.asp>]
5. CPRD Practice and Patient Confidentiality [https://www.cprd.com/researchpractice/researchgppractice.asp]
6. Data Access Request Service (DARS) [<http://content.digital.nhs.uk/DARS>]
7. Diagnostic Imaging Dataset (DIDS) [<https://www.england.nhs.uk/statistics/statistical-work-areas/diagnostic-imaging-dataset/>]
8. Database of Instruments for Resource-Use Measurement (DIRUM) [[www.dirum.org](http://www.dirum.org)]
9. EMIS Web [<https://www.emishealth.com/products/emis-web/>]
10. General Practice Extraction Service (GPES) [<http://content.digital.nhs.uk/gpes>]
11. GP Connect [[http https://digital.nhs.uk/article/1275/GP-Connect](http://systems.digital.nhs.uk/gpsoc/interface/gpconnect) ]
12. Hospital Episode Statistics (HES) [<http://content.digital.nhs.uk/hes>]
13. Improving Access to Psychological Services (IAPT) [<https://www.england.nhs.uk/mentalhealth/adults/iapt/>]
14. Mental Health Services Data Set (MHSDS) [<http://content.digital.nhs.uk/mhsds>]
15. National commissioing flows [<https://www.england.nhs.uk/ourwork/tsd/data-services/commissioning-flows/>]
16. The future of the National Programme for IT as described in 2010 [<http://webarchive.nationalarchives.gov.uk/20130107105354/http://www.dh.gov.uk/en/MediaCentre/Pressreleases/DH_119293>]
17. The National Programme for IT in the NHS: an update on the delivery of detailed care records systems 2012 [<https://www.nao.org.uk/report/the-national-programme-for-it-in-the-nhs-an-update-on-the-delivery-of-detailed-care-records-systems/>]
18. NorthWest EHealth Linked Database System [<http://nweh.co.uk/products/linked-database-system>]
19. QResearch overview [<http://www.qresearch.org/SitePages/Home.aspx>]
20. QResearch Information for Patients [<http://www.qresearch.org/SitePages/Information%20for%20patients.aspx>]
21. Read Codes [https://digital.nhs.uk/article/1104/Read-Codes]
22. ResearchOne overview [<http://www.researchone.org/>]
23. ResearchOne. 5 Million Records Support Research in 2013 [<http://www.researchone.org/400-organisations-join-in-2013/>]
24. Secondary Uses Service (SUS) [<http://content.digital.nhs.uk/sus>]
25. SNOMED CT [https://digital.nhs.uk/snomed-ct]
26. SNOMED CT in primary care [https://digital.nhs.uk/SNOMED-CT-implementation-in-primary-care]
27. SystmOne by TPP (The Phoenix Partnership) [<https://www.tpp-uk.com/products/systmone>]
28. The Health Improvement Network (THIN) overview [ <http://www.inps.co.uk/vision/thin/about-us>]
29. Vision by INPS (In Practice Systems) [<http://www.inps.co.uk/vision>]
